# Effects of summer weather and heatwaves on wild boar activity

**DOI:** 10.1098/rsos.242208

**Published:** 2025-07-23

**Authors:** Justine Güldenpfennig, Niccolò Fattorini, Miloš Ježek, Kevin Morelle, Tomasz Podgórski

**Affiliations:** ^1^ Department of Game Management and Wildlife Biology, Czech University of Life Sciences in Prague Faculty of Forestry and Wood Sciences, Praha, Czech Republic; ^2^ Department of Life Sciences, University of Siena, Siena, Italy; ^3^ National Biodiversity Future Center, Palermo, Italy; ^4^ Department of Migration, Max Planck Institute of Animal Behavior, Radolfzell, Germany; ^5^ Mammal Research Institute, Polish Academy of Sciences, Białowieża, Poland

**Keywords:** behaviour, biologging, climate change, heat, *Sus scrofa*, telemetry

## Abstract

Climate change threatens wildlife species, negatively affecting their fitness through environmental change, such as through increased severity of droughts and summer heatwaves. Wild boar (*Sus scrofa*), a species with limited physiological thermoregulation abilities, is potentially vulnerable to high temperatures during summer. Yet little is known about the behavioural reactions of this species to heat stress. Detailed understanding of wild boar behavioural adaptations to their environment might help understand their future population growth and change in the geographical range. We used multisensory collars on 24 individual wild boars in the Czech Republic, calculating the dynamic body acceleration as a proxy for energy expenditure to detect activity changes in response to high temperatures on two temporal scales (daily and seasonal) and during heatwaves. Our results revealed that overall, under higher temperatures, wild boars reduce their activity, unless it rained. Heatwave duration did not affect wild boar activity. We show that wild boars adapt their activity to weather conditions and highlight the importance of sufficient precipitation for thermoregulation in this species. This suggests that studies about climate change impacts on wildlife behaviour should consider not only rising temperatures but also shifts in rainfall patterns. Additionally, this research shows the potential of remote-sensing technologies to monitor wildlife behaviour, particularly in challenging observational scenarios, offering valuable insights into the behavioural responses of wildlife in the face of a changing climate.

## 1. Introduction

Globally, the temperature rise caused by climate change, coupled with shifting precipitation patterns, has resulted in more frequent, intense and prolonged heatwaves [1], as well as an increased occurrence of other extreme weather events such as wildfires, storms and floods [2]. These changes pose a severe threat to both ecosystems and their inhabitants. Therefore, it is crucial to understand the impact of climate change on wildlife behaviour and fitness. To evaluate an animal’s vulnerability to environmental change, it is essential to consider sensitivity, exposure, resilience and adaptive potential [3], including levels of behavioural plasticity or flexibility, through which individuals may cope with environmental perturbations [4].

Wild boar (*Sus scrofa*) can adapt to various environmental conditions. Fast population growth rates [5], omnivorous and opportunistic feeding habits [6], limited predation risk [7] and high tolerance towards human activities [8] contribute to this adaptive skill. They are widely distributed across almost all continents and are among the most globally dispersed wildlife species [9], making them one of the key species to study in the context of climate change. Wild boar are suggested to be one of the species benefitting from climate change. Winter severity was the main factor limiting wild boar population growth and density in the past [10–13]. However, with milder winters, increasing mast frequency, intensification of crop production and diminishing role of hunting, wild boar populations are constantly growing [10,14–16]. As a result of decreasing climate harshness, among other factors, wild boar populations are continuously expanding into northern countries [17]. Yet little is known about the susceptibility of wild boar to high temperatures during summer which, given its restricted physiological thermoregulation abilities [18], may soon become a population stressor, particularly in southern regions of the species’s range, such as Africa, southern Europe or even central Europe. Animals exhibit various thermoregulatory systems, including sensible heat loss (e.g. conduction, convection and radiation) and evaporative heat loss (e.g. sweating and panting) [19,20]. Wild and domestic pigs face challenges in thermoregulation due to the relatively low number of sweat glands and a thick layer of adipose tissue [21]. Studies on domestic pigs have demonstrated that during higher temperatures, they reduce their activity, increase resting phases and wallowing time, decrease feed intake and shift feeding periods to cooler parts of the day [22–25].

Previous research has documented spatiotemporal behavioural changes due to rising ambient temperatures in other wild ungulate species. These changes include reduced activity during daylight hours (moose *Alces alces* [26]; Alpine ibex *Capra ibex* [27]; springbok *Antidorcas marsupialis*; eland *Tragelaphus oryx* [28]), increased activity and feeding behaviour during early or late hours of the day (moose [29]; Alpine ibex [30]; Apennine chamois *Rupicapra pyrenaica* [31]), overall decreases in feeding time (black wildebeest *Connochaetes gnou* [32]) and the selection of thermally sheltered stands or bedding sites (roe deer *Capreolus capreolus* [33]; moose *Alces alces shirasi* [34]). Studies considering season and weather effects on wild boar focused mainly on movement analysis (e.g. travel distances [35]; daily ranges [36,37]) or activity proxies (activity tracking [38]; binary activity [39]; activity rate [40]).

Traditional methods of studying the response of large wildlife species to disturbances primarily rely on GPS-telemetry-based movement analysis [41]. However, with technological advancements, remote-sensing biologging has emerged as a promising approach to collect more detailed data on animal behaviour [42–44]. One such method is measuring motion-linked energy expenditure using acceleration [45]. Acceleration provides movement intensity, frequency and duration indicators and is easily applied in laboratory and field settings [46]. The sum of the three acceleration vectors (*xyz*), known as dynamic body acceleration (DBA), can be used to estimate movement-based power and serves as a proxy for oxygen consumption and, therefore, energy expenditure [47,48]. Whereas GPS-based activity measurement relies on actual movement in the form of travelling, DBA is able to measure the actual motion of the animal and indicate more accurate changes in activity in response to various environmental or health status stimuli [49]. Within these relationships, the vectorial sum of dynamic body acceleration (VeDBA), calculated using the vectorial sum from all three spatial axes (surge, heave and sway), and the overall dynamic body acceleration, calculated by summing the absolute values of the dynamic acceleration, show a very high correlation [47,48]. However, the VeDBA avoids interferences by small movements of the collar on the animal’s neck [47].

In this study, we used multisensory collars (combining GPS, accelerometer and magnetometer) to investigate the response of free-roaming wild boars in a suburban forest near Prague, Czech Republic, to summer temperatures, precipitation and prolonged periods above the thermoneutral zone (i.e. heatwaves). Specifically, we aimed to use acceleration-based DBA to accurately examine the change of activity of wild boars in reaction to higher temperatures at daily and seasonal temporal scales as well as within heatwaves. We tested the following hypotheses: (i) wild boar, similarly to domestic pigs, will reduce physical activity in response to increasing temperature (both within day and across the summer season); and (ii) prolonged exposure to elevated temperatures (heatwaves) without rain will induce a greater reduction in wild boar activity, highlighting the cumulative effect of heat stress over extended periods.

## 2. Material and methods

### 2.1. Climate and study area

The Czech Republic is in a moderate continental climate zone, with annual average temperatures between 1.1°C and 9.7°C (hottest months July and August; summer average 16°C/17°C [50]). The average annual temperature increased by 1.9°C between the average measured for the period 1961−1990 and the temperature measured in 2015 [47]. The annual summer (June–August) temperatures of the whole Czech Republic averaged 18.3°C during our study period (2019−2021), compared with 17.3°C for 2009−2011 and 16.9°C for 1999−2001 [51] suggesting an increasing trend in temperatures (electronic supplementary material, figure S1). In the same periods, precipitation increased from 90.3 mm for 1999−2001 to 102.1 mm and 144.0 mm for the periods 2009−2011 and 2019−2021, respectively [51] (electronic supplementary material, figure S1). The study area is located about 50 km east of Prague in the Central Bohemian Region of the Czech Republic (49.91−50.01° N, 14.68−14.88° E). The forest covers almost 7000 ha and is framed with farmland and settlements. It is a popular area for outdoor recreational activities, including walking and cycling [52].

### 2.2. Animal handling and data collection

Wild boars were trapped using a box cage or wooden corral traps that were baited with corn. After capture, they were immobilized using a tranquilliser rifle and darts containing a mixture of Zoletil, Ketamine and Xylazine (dose dependent on size/weight of the individuals [53]). Once sedated, wild boars were sexed, aged and equipped with GPS collars (Vectronic Aerospace) with attached daily diary tags (3-axis accelerometer with storing frequency 10 Hz, 3-axis magnetometer with storing frequency 10 Hz; Wildbyte Technologies [54]). The anaesthetized wild boars were left at the capture location and monitored until they regained consciousness. This work was carried out per the guidelines of the Ministry of the Environment of the Czech Republic. The trapping and handling protocol was approved by the ethics committee of the Ministry of the Environment of the Czech Republic and carried out following the decision of the ethics committee of the Ministry of the Environment of the Czech Republic, number MZP/2019/630/361. The daily diary tags recorded acceleration and magnetic heading as 10 Hz data (10 points per second), which was stored on a microSD card on the collar. After retrieval of the collar postmortem or using a drop-off system, the data could be read and stored until further processing.

### 2.3. Data processing

The smoothed sum of VeDBA was calculated using the centred rolling average over 2 s and exported using DDMT software (Wildbyte Technologies). The remaining data processing was performed within the R environment (R v. 4.2.2). Wild boars are predominantly nocturnal within our study area [52,55]. Therefore, we defined a day to start at sunrise on day *d* and end with the following sunrise on day *d +* 1 (as opposed to a midnight-to-midnight day definition). This allows us to include daylight hours (sunrise to sunset) as the resting period and the following night-time hours (sunset to sunrise) as the active period of wild boar within one day. We summarized the smoothed VeDBA to hourly sum using the *collapse* package [56] and further calculated the daily average and the mean daily average within the heatwaves. Hourly weather data were acquired using the Visual Crossing weather query builder (https://www.visualcrossing.com), which calculated the mean between the closest weather stations with available data. Heatwaves for wild boars were defined based on information about the thermoneutral zone in domestic pigs and wild boars. Studies in domestic pigs indicate optimal temperatures for farm animals around 24°C [23,57,58]. Ruf *et al.* found the summer thermoneutral zone of wild boars to be 6–24°C [18]. Therefore, we defined heatwaves as any period of 23+ h with a daily average temperature above 24°C. GPS locations were filtered by dilution of precision (DOP) ≥1 and ≤7. We calculated three daily movement metrics based on GPS locations: the daily distance travelled, the maximum daily net squared displacement and the daily average speed. Movement parameters were calculated using the *amt* package [59].

### 2.4. Statistical analysis

All statistical analyses were performed within the R environment (R v. 4.2.2), using the packages *mgcv* [60] and *glmmTMB* [61]. The dataset was restricted to observations recorded during the summer months (June to August), spanning 2019–2021, and encompassing data from 24 individuals (electronic supplementary material, table S2). Using generalized additive mixed models (GAMMs) and generalized linear mixed models (GLMMs), we analysed the effects of climatic variables (temperature and precipitation) and time (hour of the day, day of the year and duration of heatwave) on the VeDBA. Since we observed a lot of days without any precipitation (42% of the whole data set), total precipitation was expressed as a binary factor (> 0 mm ‘presence’; = 0 mm ‘absence’) in the respective temporal scales. Before building the models, we checked for correlation between the predictors. We added predictors according to the ‘forced entry’ method, including all predictors based on our hypotheses.

In the first GAMM (GAM1) assessing the daily effect of summer weather, the hourly smoothed sum of VeDBA served as the response variable, while hourly temperature, hour of the day and the interaction between temperature and hour were included as fixed effects. The binary effect of precipitation for each hour was added as a factor-smooth interaction (variable ‘by’ factor) in all smooths and added as a linear main effect. The temperature was modelled using the default thin plate regression splines, while the hour was modelled as a cyclic cubic spline function to account for its periodicity. Within the GAMM, we used the ‘*ti*’ function to define the tensor product interaction between temperature and hour while including the main effects [60]. The second GAMM (GAM2) testing the effects of summer weather at a seasonal scale was built with the daily average of VeDBA as the response. Daily maximum temperature, day of the year (DOY) and the interaction between them were set as the predictors. The interaction was added as in GAM1 using the ‘*ti*’ function, while both main effects were modelled as thin plate regression splines (no circularity occurs in DOY since our study period is restricted to summer). Precipitation presence during a day was added as a factor smooth interaction and a linear main effect. To test whether seasonal variation in the VeDBA can be explained by animal movements, we ran the model of seasonal activity with different movement parameters as the response variables. The models were structured like GAM2, using the daily maximum temperature, DOY and the interaction between them as fixed effects. Animal ID was set as a random effect, and we used the Gaussian distribution (link = identity). We focused on the effect of the DOY on movement parameters. In all GAMMs, restricted maximum likelihood (REML) was used, response variables were modelled through Gaussian errors (link: identity) and animal ID was included as a random effect to account for repeated measurements on the same individual. To ensure the robustness of the models, we used the ‘*gam.check*’ function within the *mgcv* package, confirming the adequacy of the chosen models. We inspected and quantified the results of the models using the ‘*predict*’ function of the same package.

To test for the effect of heatwaves, the average daily mean of VeDBA (response variable), average daily maximum temperature and total precipitation were calculated for the heatwave periods. As explained above, precipitation was then expressed as ‘present’ or ‘absent’. The dataset included 17 individuals out of the original 24 (electronic supplementary material, table S2). Contrary to the previous analyses, preliminary data exploration ruled out the nonlinear effects of predictors on the response variable; therefore, we used a GLMM. In the model, we included the main effects of maximum temperature, heatwave length, precipitation, the interaction between the length of heatwaves in days with the binary precipitation and the interaction between the average maximum temperature and the precipitation as fixed effects. We used Gaussian distribution (link: identity) to model the response variable and included animal ID as a random effect. The covariates were scaled to improve model convergence. Residuals of the GLMM were visually inspected using the ‘DHARMa’ package [62], and no deviation from model assumptions was detected. We also calculated marginal and conditional *R*^2^ according to Nakagawa *et al.* to assess the model’s goodness of fit [63].

## 3. Results

### 3.1. Climatic conditions

During the study period (2019−2021), annual summer (June−August) temperatures in our study area averaged 19.6°C. The hottest year was 2019, with a summer average temperature of 20.8°C (9.3–36.7°C; average daily maximum of 26.3°C), whereas temperatures in 2020 and 2021 averaged 19.1°C (8.1–33.2°C, average daily maximum of 24.0°C) and 19.0°C (5.5–32.0°C, average daily maximum of 23.4°C), respectively (electronic supplementary material, figure S2). The hottest month in 2019 and 2021 was June, whereas in 2020 the hottest month was August. Averaged over the whole study period, the daytime temperature was 20.9°C and 17.1°C during the night (electronic supplementary material, table S1). The highest precipitation occurred in 2020 (total precipitation 288.27 mm, mean precipitation 0.14 mm and 58 days with precipitation) compared with 2019 (total precipitation 174.94 mm, mean precipitation 0.08 mm and 47 days with precipitation) and 2021 (total precipitation 269.20 mm, mean precipitation 0.13 mm and 55 days with precipitation) (electronic supplementary material, figure S2). During the study period, we registered 14 sequences of a heatwave, of which 7 lasted longer than a day. The heatwaves had an average duration of 1.88 days (1–4 days, *n* = 8) in 2019, 1.25 days (1–2 days, *n* = 4) in 2020 and 3.00 days (1–5 days, *n* = 2) in 2021. Detailed information about the climate data can be found in the electronic supplementary material.

### 3.2. Daily activity patterns

**Figure 1.**
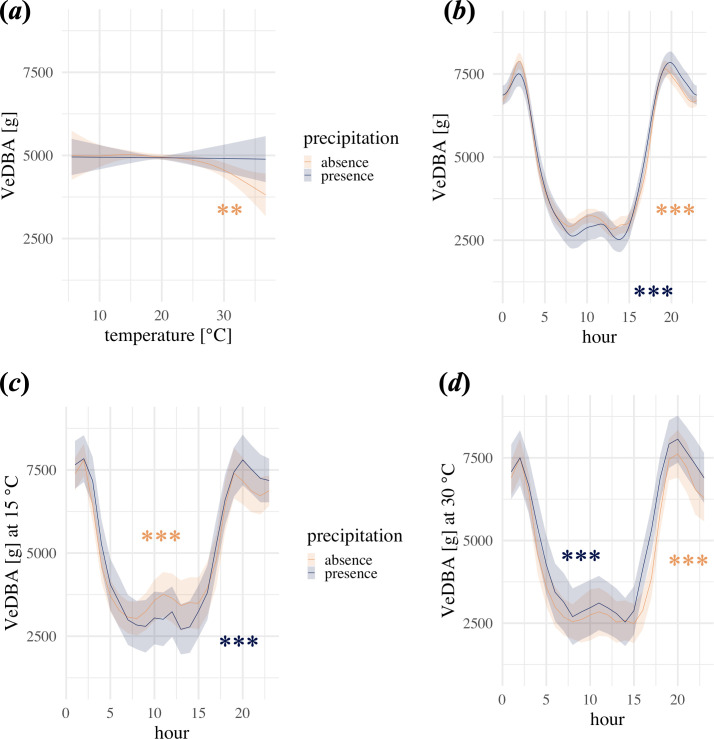
Effect plots of GAM1 visualizing the effects of temperature and hour of the day on the hourly sum of VeDBA. (*a,b*) depict the main effects of temperature and hour grouped by precipitation presence (blue) and absence (orange). The lines are the model estimates, and shaded areas represent the model 95% confidence intervals. (*c,d*) show the VeDBA in interaction with hour of the day at 15°C and 30°C, respectively. Asterisks represent significance levels (***p < 0.001; **p < 0.01; *p < 0.05) when precipitation is present (blue) or absent (orange). GAM, generalized additive model; VeDBA, vectorial sum of dynamic body acceleration.

An overview of the raw data can be seen in electronic supplementary material, figure S3. In our model of daily activity, there was a significant negative effect of temperature when precipitation was absent, whereby the VeDBA appeared to decrease exponentially with higher temperatures (figure 1*a*; table 1). When precipitation was present, temperature had no significant effect (figure 1*a*). The VeDBA was significantly influenced by the time of day and generally highest during the night, regardless of the occurrence of precipitation (figure 1*b*; electronic supplementary material, figure S4; approximately 60% higher between 22.00 and 04.00 compared with between 05.00 and 21.00). The analysis of the interaction between temperature and hour of the day revealed a significant effect, indicating that the relationship between temperature and activity varied across different times of the day (figure 1*c,d*). The difference in the VeDBA between afternoon and around sunset was larger when temperatures were high than when compared with lower temperatures (figure 1*c*). For example, at lower temperatures (15°C) with no precipitation, the VeDBA increased from the late afternoon (between 16.00 and 18.00) to around sunset (20.00 and 22.00) by 37%, whereas at warmer temperatures (30°C) with no precipitation, the VeDBA increased by 70% (figure 1*c*). When rain was present, higher temperatures had less influence on the VeDBA. Here, the late afternoon VeDBA increased by 44% to around sunset at 15°C, whereas at 30°C the increase was 41% (figure 1*d*).

**Table 1.** Detailed summary of the generalized additive model used to predict the effects of daily temperature and time on the hourly vectorial sum of dynamic body acceleration (VeDBA). In the table, ‘precip’ stands for precipitation presence or absence, ‘temp’ stands for temperature (measured hourly), ‘hour’ stands for hour of the day and ‘animalID’ stands for ID for each individual, which was added as a random effect. The predictors were included as smooth terms on their own and as an interaction between temperature and hour of the day. A factor interaction with precipitation presence or absence was added to all predictors. s.e., standard error.; edf, effective degree of freedom; ref.edf, reference edf; *n*, number of observations; GAM, generalized additive model.

		GAM1				
component	term	estimate	s.e.	*t*-value	***p*** **‐value**	
*parametric coefficients*	(intercept)	4930.346	259.245	19.018	**0.0000**	***
	precip_presence	128.780	78.848	1.633	0.1024	

component	term	edf	ref.edf	*F*-value	***p*** **‐value**	
*smooth terms*	s(temp):precip_absence	3.228	3.915	3.985	**0.0060**	**
	s(temp):precip_presence	1.023	1.045	0.008	0.9673	
	s(hour):precip_absence	17.655	22.000	162.594	**0.0000**	***
	s(hour):precip_presence	12.261	22.000	67.765	**0.0000**	***
	ti(hour,temp):precip_absence	44.592	415.000	0.253	**0.0000**	***
	ti(hour,temp):precip_presence	26.436	305.000	0.249	**0.0000**	***
	s(animalID)	21.955	23.000	94.232	**0.0000**	***

Significance levels: ***p < 0.001; **p < 0.01; *p < 0.05.

Adjusted *R*^2^: 0.294, deviance explained 0.297.

*n* = 26 400.

### 3.3. Seasonal activity patterns

**Figure 2.**
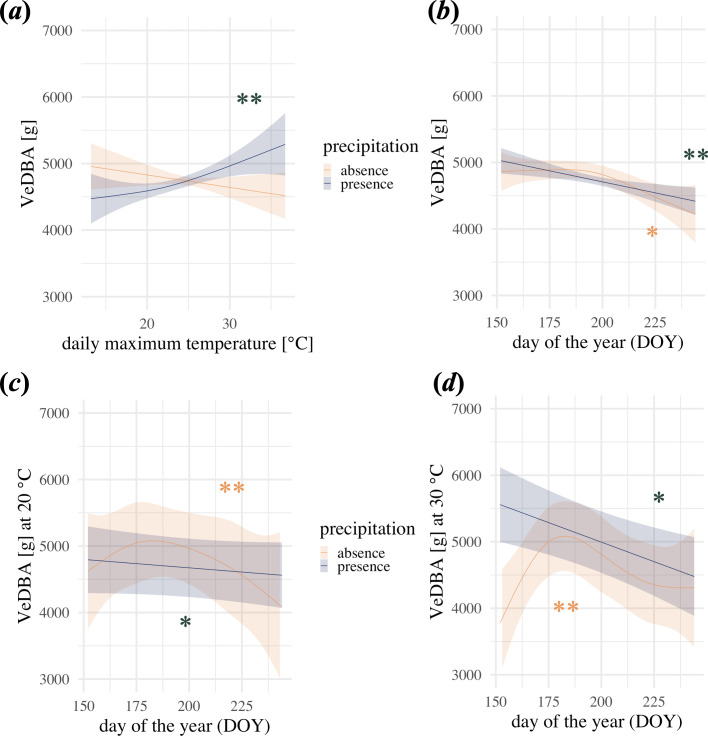
Effect plots of GAM2 visualizing the effect of daily maximum temperature and day of the year on the daily average VeDBA. (*a,b*) depict the main effects of daily maximum temperature and day of the year (DOY) grouped by precipitation presence (blue) and absence (orange). The lines are the model estimates and shaded areas represent the model 95% confidence intervals. (*c,d*) show the daily average VeDBA in interaction with day of the year at 20°C and 30°C, respectively. Asterisks represent significance levels (***p < 0.001; **p < 0.01; *p < 0.05) when precipitation is present (blue) or absent (orange). GAM, generalized additive model; VeDBA, vectorial sum of dynamic body acceleration.

In our model of seasonal activity, the VeDBA was positively related to daily maximum temperature when precipitation was present, while a negative, yet statistically insignificant, trend was observed when precipitation was absent (figure 2*a*; table 2). Throughout the summer, wild boar activity decreased, independent of precipitation (figure 2*b*). The effect of interaction between daily maximum temperature and DOY by precipitation was also significant, although the pattern differed between days with and without precipitation (figure 2*c,d*). For example, at 20°C, on days when precipitation was absent, the daily average VeDBA only increased by 2% from June to July but decreased by 10% from July to August. At 30°C, the VeDBA increased by 6% from June to July and decreased by 10% from July to August. On days when precipitation was present, the daily average VeDBA decreased by 2% between June and July and 2% between July and August at 20°C, but decreased by 7% between June and July and then decreased by 7% from July to August at 30°C.

**Table 2.** Detailed summary of the generalised additive model used to predict the effects of seasonal temperature and time on the daily vectorial sum of dynamic body acceleration (VeDBA). In the table, ‘precip’ stands for precipitation presence or absence, ‘T_max’_ stands for the daily maximum temperature, ‘DOY’ stands for day of the year, and ‘animalID’ stands for ID for each individual, which was added as a random effect. The predictors were included as smooth terms on their own and as an interaction between daily mean temperature and day of the year. A factor interaction with precipitation presence or absence was added to all predictors. s.e., standard error; edf, effective degree of freedom; ref.edf, reference edf; *n* number of observations.

		GAM2				
component	term	estimate	std error	*t*-value	*p*‐value	
*parametric coefficients*	(intercept)	4745.987	280.492	16.920	**0.0000**	***
	precip_presence	97.540	70.324	1.387	0.1657	

component	term	edf	ref.edf	*F*-value	*p*‐value	
*smooth terms*	s(*T*_max_):precip_absence	1.003	1.006	1.600	0.2064	
	s(*T*_max_):precip_presence	1.563	1.945	6.065	**0.0058**	**
	s(DOY):precip_absence	2.190	2.691	3.192	**0.0252**	*
	s(DOY):precip_presence	1.000	1.001	9.998	**0.0016**	**
	ti(DOY,*T*_max_):precip_absence	7.391	9.152	2.419	**0.0098**	**
	ti(DOY,*T*_max_):precip_presence	1.009	1.018	4.884	**0.0268**	*
	s(animalID)	22.120	23.000	38.158	**0.0000**	***

Significance levels: ***p < 0.001; **p < 0.01; *p < 0.05.

Adjusted *R*^2^: 0.482, deviance explained 0.499.

*n* = 1118.

**Figure 3.**
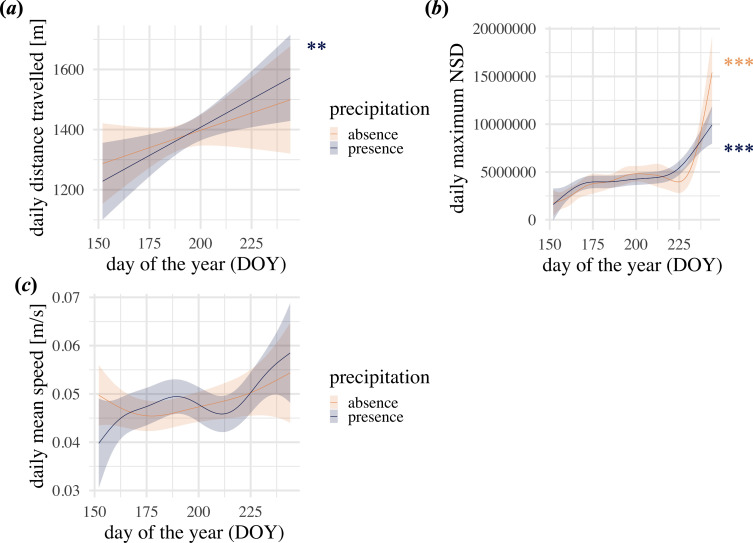
Results of the post-hoc test investigating the effect of day of the year (DOY) on movement parameters. (*a*) shows the effect of DOY on the daily distance travelled, (*b*) shows the pattern of the daily maximum net squared displacement (NSD) and (*c*) shows the pattern of the daily mean speed. Precipitation is either absent (orange) or present (blue). The lines are the model estimates and shaded areas represent the model 95% confidence intervals. Asterisks represent significance levels (***p < 0.001; **p < 0.01; *p < 0.05) when precipitation is present (blue) or absent (orange).

The models of seasonal animal movement revealed a significant continuous increase of daily distance travelled throughout the summer when it was raining (figure 3*a*). The same trend was visible on days without rain, although non-significant (electronic supplementary material, table S3). The maximum net squared displacement slightly increased from June to July and then increased steeply at the end of summer (electronic supplementary material, figure Sb). This pattern was significant with and without precipitation (electronic supplementary material, table S3). The effect of DOY on the average speed did not reveal a significant effect (electronic supplementary material, table S3), although the visual inspection revealed a slight increase throughout the summer (figure 3*c*).

### 3.4. Activity during heatwaves

We found no significant effect of heatwave length, the average daily maximum temperature or their interaction with precipitation (table 3).

**Table 3.** Detailed summary of the generalized linear mixed models used to examine the effect of heatwave intensity on the vectorial sum of dynamic body acceleration (VeDBA). In the table, predictor variables are indicated as ‘precip’ standing for precipitation presence or absence, ‘heatwave length’ indicating the number of days >24°C and ‘*T*_max_ mean’ standing for the mean daily maximum temperature within one heatwave. All predictors were added in interaction with precipitation presence of absence. CI = confidence intervals.

	GLM		
predictors	estimates	CI	*p*‐value
(intercept)	87.26	−14168.16–14342.69	0.990
precip_presence	−24.82	−16146.76–16097.11	0.998
heatwave length	−358.96	−1626.67–908.76	0.579
*T*_max_ mean	158.68	−323.25–640.61	0.519
heatwave length:precip_presence	472.09	−821.05–1765.23	0.474
*T*_max_ mean:precip_presence	−9.84	−545.63–525.95	0.971

Significance levels: ***p < 0.001; **p < 0.01; *p < 0.05.

Marginal *R*^2^/conditional *R*^2^ 0.051/0.601.

## 4. Discussion

In this study, we showed the effect of summer temperatures and precipitation on wild boar activity using high-resolution multisensory collars. Consistent with our hypothesis, when summer temperatures are higher, wild boar reduced their daily activity level. This effect was constant across the day and the summer season, counteracted by the presence of rain. Surprisingly, we found no significant effect of heatwave intensity on wild boar activity (table 3).

### 4.1. Effect of rain on reduced activity at high temperatures

We showed that wild boar activity decreased within hot and dry days and at higher daily maximum temperatures but increased when it did rain (figures 1*a* and 2*a*; electronic supplementary material, figure S3). This finding may highlight the importance of rain for thermoregulatory behaviour. Wild boars, lacking sweat glands, have to rely heavily on other mechanisms to ensure evaporative cooling, including wallowing [25,64]. A humid environment enhances evaporative cooling. Rain may directly affect cooling by wetting the skin and indirectly provide wetter ground/water holes and increase wallowing activity. This increase in body motion might also be produced by increased shaking and rolling of the body in the mud. Additionally, wet ground makes it easier for the wild boars to dig and search for food, which could be an additional explanation for the increased activity when it rains [65]. More detailed research, including in-depth behavioural analysis combined with *in situ* mapping of ephemeral water sources, are needed to test this explanation. When it was dry, wild boars seemed to reduce physical activity to prevent increasing body temperature and a resulting need for thermoregulatory energy expenditure [66]. These results are consistent with the results found in domestic pigs [25]. Reduced activity in response to hotter days was also found in other ungulates, for example mouflon *Ovis gmelini musimon × Ovis sp*. [67], greater kudu *Tragelaphus strepsiceros* [68], springbok and eland [28].

### 4.2. Temporal effects on activity in interaction with the weather

The temporal pattern of body motion (measured with the VeDBA) found in our study showed primarily nocturnal activity, consistent with previous studies [38,39,69–71] that found that human activities drive the diel activity cycle in this species. At the same time, studies in other ungulates found that temperature might be a more significant factor driving nocturnality than human pressure (e.g. in northern chamois *Rupicapra rupicapra* [72]) or predation risk (e.g. in alpine ibex [73]). Therefore, temperature may be an important factor in the evolution of nocturnality, although it is not yet clear how big this effect is on wild boars.

Although overall night activity was higher than during the day, our results showed two acrophases at dusk and dawn. The activity peaks at dusk and dawn might be caused by actual locomotion behaviour from resting sites to other areas of activity. The short decrease in activity detected from midnight until dawn might be caused by feeding behaviour. Feeding behaviour is slower [74] than travel movement. DBA was found to increase with speed [24,75], suggesting a higher VeDBA during travel movement (including trotting and running) than in comparison to slower feeding/rooting movement (only including slow walking). Additionally, our results showed that the peak of the VeDBA at sunset appeared higher on days with hotter temperatures than on days with lower temperatures (figure 1*c*). We suggest this might be caused by an interaction of decreasing temperatures after sunset, the search for water sources, feeding activity and the period available for activity due to short nights. Wild boars are likely to maximize the efforts of their active phase while minimizing the duration of movement, essentially increasing their activity for a shorter period than on milder nights. This highlights that wild boar are able to display behavioural flexibility in their daily activity patterns in response to environmental conditions including temperature. The increase in nocturnal activity of wild boars in response to high maximum temperature was previously shown by Brivio *et al.* [40]. A similar effect of maximum temperature in warmer months of previous diurnal hours on nocturnal activity was also found in ibex [73], free-ranging sheep (*Ovis aries*) [76] and reindeer (*Rangifer tarandus tarandus*) [77]. The strong increase of activity at sunset after hot days could also be explained by food availability. Most vertebrates and invertebrates, which are part of wild boars’ diet, are likely to be inactive during the day when temperatures are high and become active at sunset.

In our data, wild boars decreased their overall activity from June to August (figure 2*b*). However, when looking at the seasonal effect (day of the year) combined with the maximum daily temperature and precipitation, it became evident that there were subtle differences. When it was dry, wild boars slightly increased their activity from June to July but then decreased their activity from July to August. The slight increase was marginally higher during hotter days. When it did rain there was a continuous decrease of activity throughout the whole season, which intensified when it was hotter. Harvest of crop fields surrounding our study area usually started in mid-July and finished in August. Wild boars use crop fields for feeding and if enough shelter is provided also as all-day habitat [78]. Before the harvest, the increased activity from June to July might have resulted from movement between the forest and crop fields, especially since crops ripen and provide an attractive food source [79,80]. The harvest in July and August caused a decline in overall wild boar activity because of the loss of shelter (especially at higher ambient temperatures) and confinement to the forest habitat. However, when it rained, foraging behaviour within the forest habitat might have been enhanced, reducing the need for the wild boars to travel. To test this post hoc hypothesis, that the increase of activity was caused by changes in travel movement pattern, we run the model of seasonal activity with three different movement parameters as the response. We found an overall increasing trend of all three movement parameters throughout the summer (figure 3). This outcome is contrary to the observed decrease in the VeDBA over the summer. In conclusion, the seasonal changes of the VeDBA cannot be explained by travel movement.

We could not find an effect of heatwave intensity (length and average daily maximum temperature) on wild boar activity. It seems possible that during the heatwaves according to our definition, temperatures fluctuated enough resulting in colder nights that cancelled the effect of hotter daytime temperatures. Wild boars seemed to be able to find enough thermal shelter as well as water resources to mitigate the effects of hotter periods. This becomes evident when simultaneously looking at the results from our first model. On the other hand, we might not see an effect due to the small number of defined heatwaves (*n* = 14). Small sample sizes may cause a lack of precision and statistical power.

While the wild boars in our study area were indeed negatively affected by increasing temperature, we observed a change of the distribution of activity bouts to colder hours of the day. With future development of climate change, we might be able to see significant changes, especially if the overall increase of temperature results in tropical nights (temperatures do not fall under 20°C) or longer periods of high temperatures and drought. For example, a study in reindeer found an overall decrease in activity throughout an extreme heatwave that lasted 21 days [77].

## 5. Conclusion

The VeDBA, derived from triaxial acceleration, was able to effectively illustrate changes in wild boar activity in great detail. Our results showed how wild boars reduced their physical activity under hotter and dryer conditions while maximizing their activity during cooler hours of the day, highlighting their ability to adapt to environmental conditions. Furthermore, our results demonstrate the importance of accelerometer data to detect fine-scale changes in behaviour, that traditional GPS relocation data are not able to capture. Taking a step further, future research might use this acceleration data to classify and quantify specific behaviours [43,81].

Under climate change, understanding behavioural flexibility and its limits is key in adapting adequate management strategies [82]. However, the effect of environmental conditions on animal physiology and behaviour remains complex. We assume that as long as wild boars find enough shelter and water resources, they will be able to cope with the heat, although this might impact species distribution (e.g. favouring their geographical range expansion to the north). However, as temperatures are continuously rising, combined with more prevalent periods of droughts [2,50] and altered rainfall patterns, even wild boar might not be able to adapt quickly enough. This will be more prevalent in the southern parts of their range. If animals cannot compensate for the reduced activity and feed intake, this might lead to a switch of the limiting season for population growth from winter to summer or at least cancel out the positive effects of climate change. This is an important issue for future research as it implies critical management implications.

## Data Availability

The data and code were submitted to Dryad [83]. Supplementary material is available online [84].
